# Supplementary material for: Reconciling Mining with the Conservation of Cave Biodiversity: A Quantitative Baseline to Help Establish Conservation Priorities
Source: PLoS One. 2016 Dec 20;11(12):e0168348. doi: 10.1371/journal.pone.0168348 (PMC5173368; doi:10.1371/journal.pone.0168348)
Supplement: S1 Dataset — (ZIP) [file pone.0168348.s002.zip › Taxa/Serra Norte/SN_2013/N4E/Lista N4E-24.pdf]

CAVIDADE N4E-0024

Classe	Ordem	Fam/Outros	Gên/Outros	Espécie	S-U	U-S	Zon.
Arachnida	Acari	(Parasitengonina)		sp.2		X	P
Arachnida	Acari	Argasidae	*Ornithodoros*	sp.1	X	X	P
Arachnida	Acari	Prostigmata		sp.2		X	P
Arachnida	Araneae	Filistatidae		sp.1	X	X	E/P
Arachnida	Araneae	Mysmenidae		jovem		X	E
Arachnida	Araneae	Pholcidae	*Leptopholcus*	sp.1		X	P
Arachnida	Araneae	Pholcidae	*Mesabolivar*	sp.1		X	E/P
Arachnida	Araneae	Pholcidae	*Metagonia*	sp.	X		
Arachnida	Araneae	Scytodidae	*Scytodes*	sp.	X		
Arachnida	Pseudoscorpiones	Chernetidae	*Spelaeochernes*	sp.1	X	X	P
Insecta	Blattodea	Polyphagidae		jovem		4	P
Insecta	Coleoptera			jovem		X	P
Insecta	Diptera	Psychodidae	*Breviscapus*	sp.		X	P
Insecta	Diptera	Sciaridae		sp.		X	P
Insecta	Diptera	Tipulidae		sp.	X		
Insecta	Heteroptera	Reduviidae	Emesinae	sp.6		1	P
Insecta	Hymenoptera	Formicidae	*Camponotus*	*crassus*	X		
Insecta	Hymenoptera	Formicidae	*Camponotus*	sp.1		X	E
Insecta	Hymenoptera	Formicidae	*Pachycondyla*	sp.2		X	E
Insecta	Hymenoptera	Formicidae	*Paratrechina*	sp.1		X	P
Insecta	Isoptera	Termitidae	*Nasutitermes*	sp.	X	X	E/P
Insecta	Lepidoptera	Noctuoidea		sp.2		2	E
Insecta	Lepidoptera	Tineoidea		jovem	X		
Insecta	Lepidoptera	Tineoidea		sp.1		X	E/P
Insecta	Lepidoptera	Tineoidea		sp.3		X	P
Insecta	Neuroptera	Myrmeleontidae		jovem	X		
Insecta	Orthoptera	Phalangopsidae	*Phalangopsis*	sp.		5	P
Amphibia	Anura	Leptodactylidae	*Leptodactylus*	cf. *vastus*	1		P
Amphibia	Anura			sp.		1	E
Mammalia	Chiroptera	Emballonuridae	*Peropteryx*	sp.	5	4	E/P
Mammalia	Rodentia	Cricetidae	*Rhipidomys*	sp.	1		P
